# Future increased risk from extratropical windstorms in northern Europe

**DOI:** 10.1038/s41467-023-40102-6

**Published:** 2023-07-22

**Authors:** Alexander S. Little, Matthew D. K. Priestley, Jennifer L. Catto

**Affiliations:** Department of Mathematics and Statistics, University of Exeter, Exeter, EX4 4QE United Kingdom

**Keywords:** Natural hazards, Atmospheric science, Climate change

## Abstract

European windstorms cause socioeconomic losses due to wind damage. Projections of future losses from such storms are subject to uncertainties from the frequency and tracks of the storms, their intensities and definitions thereof, and socio-economic scenarios. We use two storm severity indices applied to objectively identified extratropical cyclone footprints from a multi-model ensemble of state-of-the-art climate models under different future socio-economic scenarios. Here we show storm frequency increases across northern and central Europe, where the meteorological storm severity index more than doubles. The population-weighted storm severity index more than triples, due to projected population increases. Adapting to the increasing wind speeds using future damage thresholds, the population weighted storm severity index increases are only partially offset, despite a reduction in the meteorological storm severity through adaptation. Through following lower emissions scenarios, the future increase in risk is reduced, with the population-weighted storm severity index increase more than halved.

## Abstract (web summary)

Storm severity indices of European winter storms in climate models show future increased storm losses in northwestern Europe, caused by changes in the location and intensity of storms, and increasing population.

## Introduction

Across the European continent, extratropical cyclones dominate the prevailing weather conditions and are one of the most significant natural drivers of insured losses, with individual storms having the potential to cause more than €6 billion in losses^1,2^. The extreme wind speeds associated with particularly intense cyclones (‘windstorms’) can cause significant and widespread damage to buildings and utilities infrastructure^2^, and have large impacts on aviation, shipping, forestry and agricultural activities^3^. Examples of severe windstorms include storms Lothar and Martin from December 1999, which resulted in losses of 8bn and 3.3bn USD, respectively. Storm Kyril from January 2007 also caused 6.7bn USD insured losses^4^, and had total economic losses much greater than this.

There is considerable inter-annual variability of winter storms over western Europe^3^, with the frequency of storms peaking in the early 1990s, a declining trend followed this until 2014, associated with changes in the North Atlantic Oscillation^5^. These varying trends are also associated with uncertain projections of future storm tracks, with this being especially notable across the North Atlantic and for windstorms across western Europe^6,7^. Some consistent signals are a decrease over the south of Europe, including the Mediterranean, and an increase over the British Isles and parts of Northwestern Europe^8,9^. How the winds associated with the storms will change can be quantified in a number of different ways^10^. In the Northern Hemisphere winter, the wind intensity of extreme storms is expected to increase in the future^11^ despite decreases in overall storm numbers. Other studies show a decrease in the cyclones associated with strong winds across the NH but with increases over NW Europe^8^, indicating that these results often come with strong regional variations^12^. Winds associated with individual cyclones could increase in strength^13,14^, and mesoscale wind features (up to 50 km), such as sting jets, could increase in intensity^15^. These mesoscale changes seem to be related to the robust projections of increased precipitation within European extratropical cyclones^16–18^, and the associated increased latent heating^11^.

Previous examinations of potential future storm losses indicate an increase over the northwest of Europe, although with varying magnitudes^19–21^, consistent with storm track changes. A synthesis of previous work suggests that a future 2.5 °C increase in temperature could lead to a 23% increase in European windstorm losses^22^. Future losses from windstorms over Europe will depend on several factors that can be related to the IPCC framework of risk being a function of hazard, exposure, and vulnerability. The hazard here depends on the frequency of the windstorms and the intensity of the winds within the storms, the exposure depends on the track or location of the windstorms, and the population of the region (assuming that population will be proportional to the value of the insured assets) while the vulnerability depends on the degree of adaptation to extreme wind speeds in the present and future (amongst other factors not considered here).

In this study, we used eight state-of-the-art general circulation models (GCMs) participating in phase 6 of the Coupled Model Intercomparison Project (CMIP6)^23^ to assess the future changes of the five factors that determine the losses associated with winter (December, January, February; DJF) European windstorms between the recent past (1980–2010) and the end of the current century (2070–2100)^24^. Previous studies considering losses may have used only single models^21^, consider only daily maximum wind speeds^25,26^, or use previous generations of models with resolutions considerably lower than offered by CMIP6 models^19^, meaning we can provide a better estimate of uncertainty and robustness, using models that can better represent extratropical cyclone wind features^27^. We consider two different Shared Socio-economic Pathways (SSPs) forming part of the IPCC’s Sixth Assessment Cycle^24^: the first envisages policy efforts to control greenhouse gas emissions that follow current trends (SSP2-4.5)^28^ and has an end of 21st-century global mean warming of 2.6 °C, while the other envisages very high emissions driven by increased exploitation of fossil fuel resources (SSP5-8.5) with an end of 21st-century warming of over 5 °C relative to pre-industrial^29^.

To quantify storm impacts, we use two versions of a storm severity index (SSI)^30^; the meteorological SSI (METSSI), which only depends on the storm winds; and the sociological SSI (SOCSSI), which includes population data and correlates strongly and significantly with actual storm losses (Fig. S1). Other methods and models for quantifying windstorm damage have previously been implemented^25,26,31–33^; however, these either do not consider the impacts of objectively identified windstorms associated with extratropical cyclones or do not analyse historical and future climates. The SSI uses a fixed location-specific damage threshold based on a percentile of the wind speed distribution. If the climatology of winds changes in the future, the impacts of those changes will depend on whether adaptation to the changing wind distribution has occurred (e.g. changes to building codes). We, therefore, consider an idealised adaptation scenario by using varying wind/damage thresholds for the historical and future climates, finding that this can substantially impact future SOCSSI changes. We also account for potential changes in sociological factors, such as population demographics, which will interact with changes in cyclone severity to affect actual wind risk from social and economic perspectives^34^.

## Results

### Frequency and location of windstorms

**Fig. 1:**
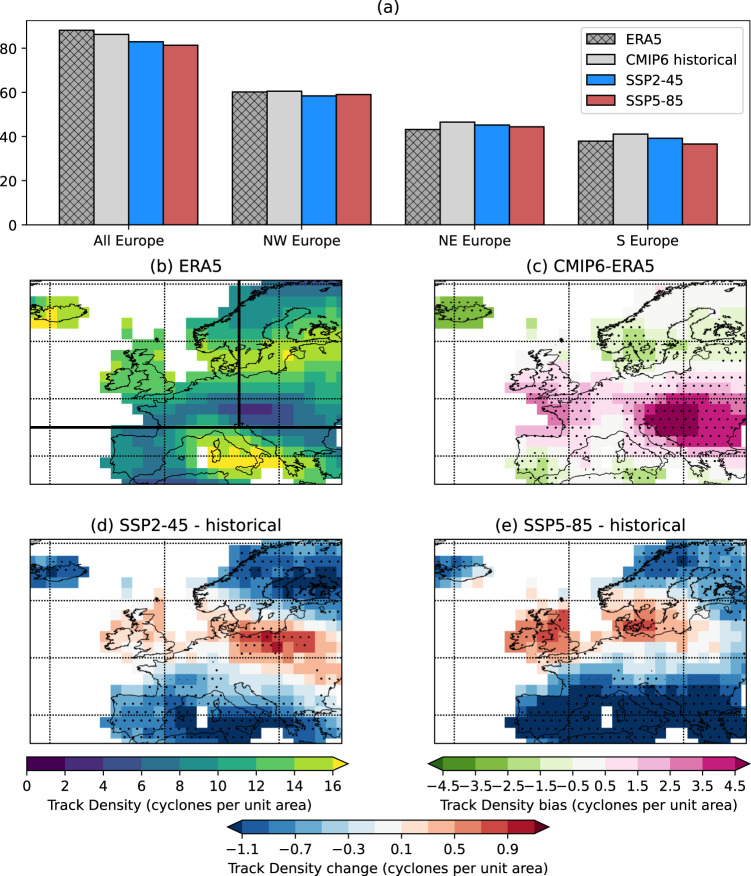
Winter cyclone frequencies. Panel (**a**) shows the total tracks per winter season for the whole European region and the three subregions (see Methods) for ERA5, CMIP6 historical, CMIP6 SSP2-4.5 and CMIP6 SSP5-8.5. The tracks are counted in each subregion they pass through, and so may be counted more than once. Panel (**b**) shows the ERA5 track density (1980–2010) with black lines indicating the geographical extent of each subregion, **c** shows the difference between CMIP6 historical and ERA5 for the same period, **d**, **e** show the multi-model mean future changes of track density in the SSP2-4.5 and SSP5-8.5 scenarios, respectively. The units of the map plots are cyclones per month per unit area. Stippling in (**c**–**e**) indicates where at least seven of the eight models agree on either the sign of the bias or the sign of future change.

There are decreases in winter cyclone frequency over Europe as a whole under both SSP2-4.5 (–4%) and SSP5-8.5 (–6%) (Fig. 1a) relative to the historical simulations. There is a strong inter-model consensus that the frequency will decrease over most of Southern Europe and Northern Scandinavia (Fig. 1d, e). In a central latitudinal band of approximately 50°N–60°N there is a small increase (up to 0.8 cyclones per month) that is subject to model uncertainty in the SSP2-4.5 simulations, and a larger, more robust increase (up to 1.2 cyclones per month) in SSP5-8.5 (Fig. 1d, e). In particular, the increases are strongest in Poland/Eastern Europe under SSP2-4.5, and the British Isles and Denmark under SSP5-8.5. The slight decrease in the NW Europe box is a result of the reduction in tracks across central and southern France (Fig. 1a), and there is also variability in the exact location of increases projected by the models (Fig. S2). The results are generally indicative of an eastward extension of winter cyclone tracks into Europe in future decades, consistent with previous studies^8,9,35–37^. The robust nature of the changes is despite the CMIP6 models strongly overestimating the baseline cyclone frequency in south-eastern Europe relative to ERA5 reanalysis (Fig. 1c), associated with recent findings suggesting that the exit of storm tracks in the model framework are too zonal^38^.

### Model cyclonic wind speeds

**Fig. 2:**
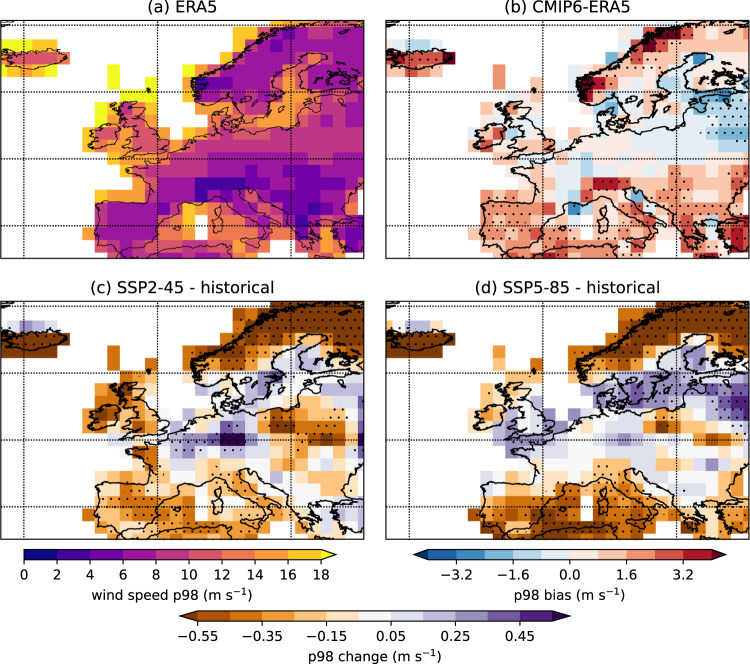
Extreme winter wind speed climatology in Europe. **a** The 98th percentile of surface wind speed from ERA5, **b** the difference between the CMIP6 multi-model mean 98th percentile of surface wind speed for the historical simulations for the period 1980–2010 and ERA5 for the same period. **c**, **d** the difference in the 98th percentile of surface wind speed for the SSP2-4.5 and SSP5-8.5 scenarios (2070–2100) compared to the historical simulations. The units are m/s. Stippling in (**b**–**d**) indicates where at least seven of the eight models agree on either the sign of the bias or the sign of future change.

**Fig. 3:**
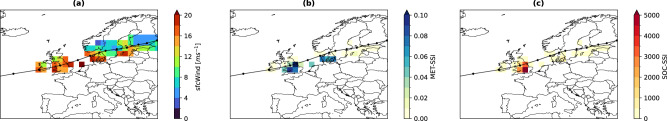
Footprints of Cyclone Daria (Burns’ Day Storm; 26/1/1990) derived from ERA5 10-metre wind speed. Panel (**a**) shows the maximum cyclonic wind speed associated with this storm as it passes over Europe. The black line with black dots shows the path of the cyclone centre. Panel (**b**) shows the Meteorological Storm Severity Index (METSSI) for the same cyclone. Panel (**c**) shows the Socio-economic Storm Severity Index (SOCSSI) for the same storm. The SOCSSI is a population-weighted severity index using the historical population estimate.

Losses from winter windstorms generally occur when the winds exceed a local threshold of the 98th percentile^30^, or 9 m/s if the 98th percentile is below that threshold^39^. Extreme wind speeds over Europe are most commonly associated with extratropical cyclones and the fronts that come with them^40,41^, which can be seen by the similar pattern of high 98th percentile winds (Fig. 2a) and high storm frequency (Fig. 1b). CMIP6 simulations overestimate the 98th percentile of the winter wind speed distribution across most areas of Europe compared to the ERA5 reanalysis, with the largest biases over coastal and mountainous areas (Fig. 2b). Projections of the 98th percentile wind speeds indicate a decrease in extreme wind threshold over most of the continent (Fig. 2c, d), with some localised increases over Germany and the Baltic Sea coasts in SSP2-4.5, and over a swath through central Europe in SSP5-8.5. The pattern of change in the 98th percentile wind (e.g. Fig. 2d) is similar to the pattern of change in the track density (Fig. 1e). Another way of analysing the extreme winds is by the maximum cyclonic wind speed associated with windstorms (Fig. 3a). Future changes in this measure for NW and NE Europe indicate a decrease in the frequency of the storms in the middle of the distribution, and not much change in the tails (Fig. S3). This suggests that the change in the 98th percentile of the wind speed is associated with the increased frequency of the storms with higher wind speeds, rather than an increase in the maximum winds the storms attain. However, it should be noted that surface wind speeds in CMIP5 models have been shown to unreasonably respond to arbitrary changes in surface properties^42^; however, we find no evidence that this is influencing our findings (Fig. S4).

### Changes in cyclone severity and risk

Having shown that parts of Europe will experience increases in the intensity of the extreme winds, we can consider two different sub-scenarios to investigate future storm severity and risk. One represents an ‘adaptation’ sub-scenario, where the local 98th percentile value used to calculate the storm severity indices (see Methods) is extracted from the considered socio-economic scenario; and a ‘no-adaptation’ scenario, where the storm severity threshold is calculated based only on the historical baseline distribution^19^. The benefit of using the local 98th percentile value of the winter wind speed distribution is that it is an indicator of the climatological resilience to extreme wind speeds at a given grid point. This approach is based on the assumption that while future increases in a locality’s extreme winter wind speed climatology would lead to ‘adaptation’ to these changes (for example, through the construction of more wind-proof buildings), any future decreases in these extremes as shown in Fig. 2 would not lead to the analogous but unrealistic effect of ‘de-adaptation’, and so the 98th percentile threshold cannot decrease in the future. To be clear, given that wind-related losses occur above the 98th percentile (i.e. on average approximately seven days per year), adapting to a future wind speed climatology does not imply that there will be no wind-related losses, but rather that adaptation or resilience will be at the same level as in the historical period.

**Fig. 4:**
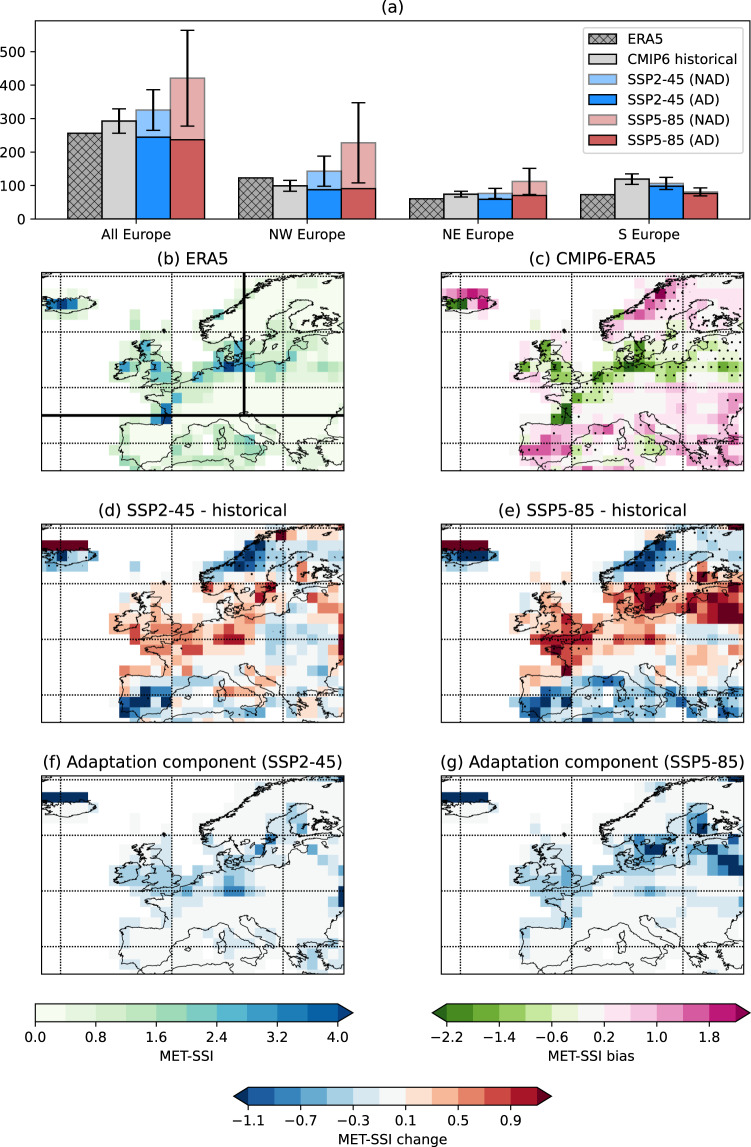
Meteorological storm severity index (METSSI) in the current and future climate. Bar chart (**a**) showing the METSSI for ERA5 (1979–2018), the CMIP6 historical simulations, and the SSP2-4.5 and SSP5-8.5 scenarios for Europe as a whole and the three subregions (see Methods). The full bars for the future simulations indicate the METSSI when the historical 98th percentile of wind speed is used as the threshold (no-adaptation case). The darker bars show the case when the 98^th^ percentile threshold is taken from the corresponding scenario (adaptation case). Error bars show the standard error of the mean for the CMIP6 models. **b** Map of the METSSI from ERA5. **c** Maps of the difference between the METSSI from the CMIP6 historical simulations and ERA5. Stippling indicates robustness between models (where at least seven of the eight models agree on the sign of bias). **d** The multi-model mean change between the future in the SSP2-4.5 scenario and the historical simulations, and (**e**) the same as (**d**) but for the SSP5-8.5 scenario. **f**, **g** The component of change associated with adaptation (i.e., using the future 98th percentile threshold). Values are non-dimensional, scaled by 10^−2^.

The accumulated storm severity indices are the sum of these indices from all tracked cyclones that occur over 30 winters (see Methods), equivalent to the annual exceedance probability^1^. First, we consider the METSSI, which is a measure of the severity of the storms in a meteorological sense only. While the CMIP6 model average METSSI is biased relative to the reanalysis (Fig. 4c), the models provide some robust signals in the future changes of the index. The 30-year accumulated METSSI for the no-adaptation case increases in Northern Europe and decreases in Southern Europe for both SSPs (Fig. 4a). For the higher warming (SSP5-8.5) scenario, these changes tend to be of the same sign but greater in magnitude relative to SSP2-4.5 (an increase for all Europe of 11.2% in SSP2-4.5 and of 43.7% in SSP5-8.5); this supports the argument that the dynamics of extratropical cyclones over Europe are strongly sensitive to atmospheric warming^9^. Changes noted in the model mean are, however, subject to considerable model spread (Fig. S5). Model variability can be a very large contributor to uncertainty in European wind projections^43^, and we find that the increase in METSSI is dominated by the large change in the BCC-CSM2-MR model. BCC-CSM2-MR has the largest increase in track density over NW Europe (Fig. S2) and one of the largest increases in the length of the tail of the wind speed distribution. Of the remaining seven models, four show a decrease in METSSI for NW Europe in the SSP2-4.5 scenario, and two models show a decrease in METSSI from SSP2-4.5 to SSP5-8.5. When we remove BCC-CSM2-MR from our model pool, this results in a negligible increase in METSSI across Europe (+0.42%), although still an increase over NW Europe (+13.3%).

Much of the increase in METSSI over Europe corresponds to locations where increases in the 98th percentile winds (Fig. 2), and increased track density (Fig. 1) are seen. When considering adaptation to increasing extreme winter wind speeds, we can see this mitigates projected increases in severity of storms (Fig. 4a, f, g), giving a decrease in the METSSI over Europe of 44% (Fig. 4a). This effect is most pronounced in NW Europe (reduction of 60%; Fig. 4f, g) where the extreme winds increase the most, is least pronounced in S Europe, and is higher for the SSP5-8.5 scenario. The decreased METSSI due to adaptation is a result of the increased threshold of the 98th percentile, therefore reducing the magnitude of exceedance of the cyclone windspeeds. A large component of the decrease is through removing the large increase in the BCC-CSM2-MR model (Fig. S5). In S Europe, it is likely that as the threshold is 9 m/s in both the historical and future period, then there are no changes to the METSSI from adaptation.

**Fig. 5:**
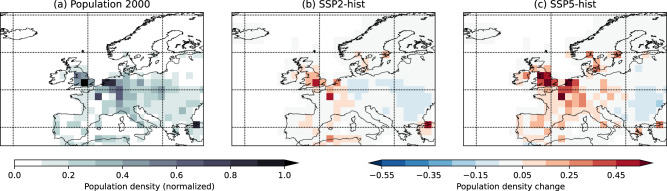
Historical and future population estimates. Values are scaled to the maximum value over the grid for the historical period. Panel **a** shows the population map used for the historical period, assuming a base year of 2000. Panels **b**, **c** show the estimated changes in 2090 relative to (**a**) under SSP2-4.5 and SSP5-8.5, respectively.

We next consider the SOCSSI, which we can use as a proxy for aggregate losses^30^, by scaling the METSSI by the historical and future population estimates (Fig. 5)^44^ at each grid point. By comparing the SOCSSI for the top 13 storms against the actual losses for these storms^4^ we see an *R*^2^ value of 0.46 (Fig. S1), indicating that there is a significant relationship between the two metrics and that the SOCSSI is a valid metric for estimating storm risk.

**Fig. 6:**
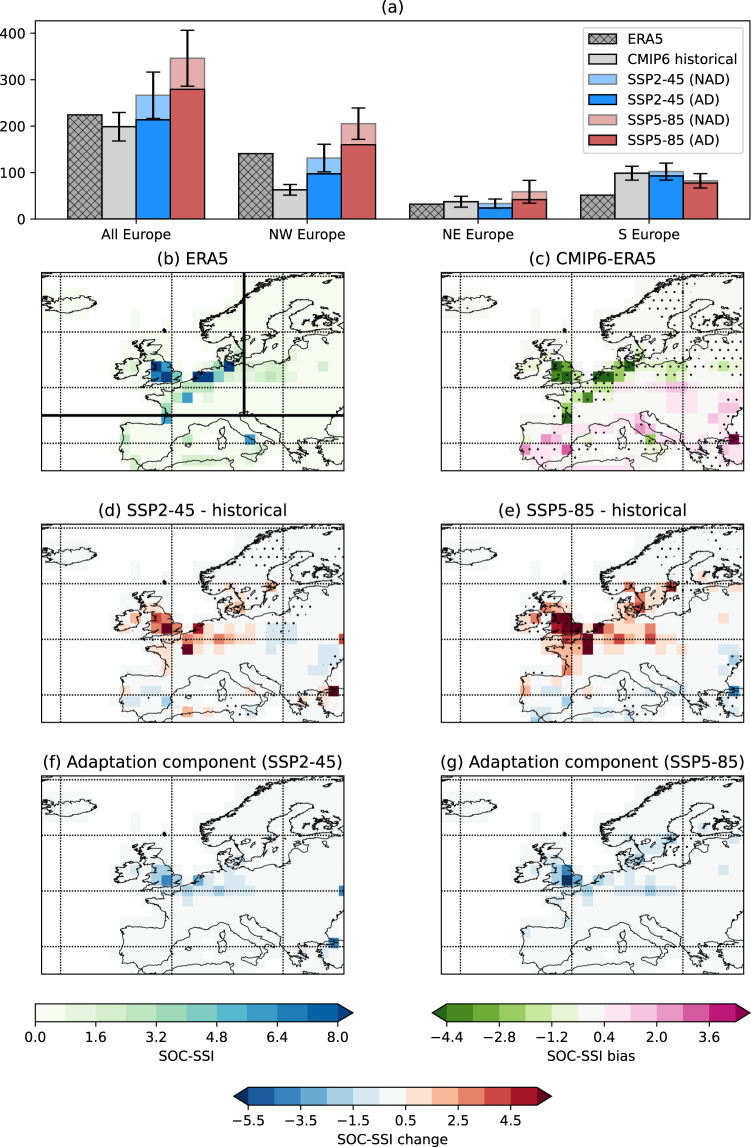
Socio-economic storm severity index (SOCSSI) in the current and future climate. Bar chart **a** showing the SOCSSI for ERA5 (1979–2018), the CMIP6 historical simulations, and the SSP2-4.5 and SSP5-8.5 scenarios for the whole of Europe and the three subregions (see Methods). The full bars for the future simulations indicate the values when the historical 98th percentile of wind speed is used as the threshold (no adaptation case). The darker bars show the case when the 98th percentile threshold is taken from the corresponding scenario (adaptation case). Error bars show the estimation of the standard error of the mean of the CMIP6 models. **b** Map of the SOCSSI from ERA5. **c** Maps of the difference between the SOCSSI from the CMIP6 historical simulations and ERA5. Stippling indicate robustness between models (where at least 7 out of the 8 models agree on the sign of the bias). **d** The multi-model mean change between the future in the SSP2-4.5 scenario and the historical simulations, and (**e**) the same as (**d**) but for the SSP5-8.5 scenario. **f**, **g** The component of change associated with adaptation (i.e. using the future 98th percentile threshold). Values are non-dimensional, scaled by 10^2^.

Figure 6 shows the SOCSSI, including the projected population change in SSP2-4.5 and SSP5-8.5. Considering first the case where no adaptation is included, we see increases in the SOCSSI are projected over Europe as a whole of 34.1% in SSP2-45 and 74.1% in SSP5-8.5 (Fig. 6a). The increases are more focused on the urban centres of NW Europe for the SOCSSI (Fig. 6a, d, e) under both SSPs, due to the population weighting, with increases both more pronounced and simulated by all of the CMIP6 models in the higher emissions scenario. Increases more than triple (+226%) for NW Europe in SSP5-8.5, with markedly less model variability than in the METSSI (Fig. S6). The large increases in METSSI noted in the BCC-CSM2-MR model is less apparent for SOCSSI due to weighting on the population centres. Population change is clearly the dominant factor accounting for more than 50% of the SOCSSI increase relative to the historical SOCSSI for W/NW Europe (Fig. S8). Over S and E Europe, changes in population density are small, and therefore the decrease in METSSI is the dominant factor in the SOCSSI changes (Fig. S8e). Where SOCSSI is increasing, changing population is the largest contributing factor and where SOCSSI is decreasing, it is because of a reduced meteorological storm intensity. However, over W/NW Europe, the SOCSSI would still increase without any change in population, due to the METSSI increases. Fig. S7 shows the distributions of METSSI and SOCSSI and indicates that the increased losses seen in the average figures will be strongly associated with increased frequency of the most extreme storms (those in the tails of the distribution; panels a–c, e–g).

The impacts of adaptation to the future wind speed threshold on the SOCSSI (Fig. 6f, g) are not as large as for the METSSI, with the largest effect being seen over the south of the UK. This means that for NW Europe, and Europe as whole, even with adaptation, there is still an increase in the SOCSSI (Fig. 6a). For the SSP2-4.5 scenario, for all of Europe, there is an increase with no adaptation of 34.1%, and for the adaptation case this increase is 7.6% relative to the historical scenario. For the SSP5-8.5 scenario, there is a no adaptation increase of 74.1%, which is only 40.5% when accounting for adaptation.

## Discussion

This study has assessed changes in the risk potential from European windstorms associated with extratropical cyclones from a multi-model perspective. Changes have been assessed for these objective features across a range of climate scenarios with varying levels of warming at the end of the 21st century. Our results strongly evidence a long-term increase in risk potential from winter windstorms in Northwestern Europe, that is more pronounced under larger warming. We find that although storm frequency is expected to decrease over Europe on average, there are local projected increases over the UK, the north of continental Europe and the south of Scandinavia. There is more than a doubling of METSSI (129% increase) from the historical value over NW Europe for the SSP5-8.5. This increase is associated with the robust location shift of the highest storm frequencies, rather than changes in the within-storm wind speeds. There is, however, considerable variability between models in their storm track and METSSI response, demonstrating the benefit of the ensemble used in this study. The multi-model changes are consistent with the findings of the latest IPCC report^7^; however, there is increasing evidence that the smaller-scale high-intensity winds will also increase, shown by cloud-permitting models^45^, which may not be accounted for here by the relatively course resolution of ~100 km.

The increased windstorm risk for NW Europe is consistent with studies using previous generation climate models^20,21,46^ and from studies assessing wind risk in CMIP6 models^43^. However, our results find markedly higher potential increases in risk, with a potential tripling of windstorm impacts across NW Europe. There are several potential reasons for this difference; internal variability; higher resolution of current global climate models; differences in climate scenarios; or the higher climate sensitivity of CMIP6 models^47^. Regarding climate sensitivity, the models we have used here consist of a broad range, with a mean that is lower than the CMIP6 all-model mean^47^. All studies agree on an increase in windstorm risk as we move through the 21st century; however, further investigation is required to determine the magnitude of this change.

The future projections of the population have been used to estimate the impact of population change on the projected losses. Despite decreases in the projected meteorological intensity of windstorms in the future with risk adaptation, increases in population by the end of the century lead to larger risk potential through an increase in socio-economic exposure. This is a feature that is robust across all of the models examined. Many of the local increases in SOCSSI concentrated across North Western Europe can be offset somewhat by adapting to increases in the extreme wind speed climatology. For the SSP5-8.5 scenario, the increase in SOCSSI is reduced by 19% (averaged over all of Europe) by including adaptation to the increased extreme windspeeds (compared increase of 74.1% with no adaptation and an increase of 40.5% with adaptation). The increased risk over Northwestern Europe in the SSP5-8.5 scenario, even when including adaptation, is more than double the historical value, indicating the adaptation is useful, but not sufficient, at reducing the future storm risk. Even if the population does not grow, adaptation to the future wind speed climatology would be necessary to avoid an increase in storm losses (i.e. the METSSI). For the more moderate climate change scenario, almost all of the population- and wind-driven increases in the impacts are mitigated by adapting to the higher wind speeds (34.1% increase with no adaptation and 7.6% increase with adaptation). So mitigating the greenhouse gas emissions, or following lower emissions scenarios, would be another way to limit the future increases in storm loss and would require less adaptation. Our approach to losses and adaptation in this study is an idealised approach and does not account for added complexities in land use change^42^ and differing vulnerabilities on a national and regional level. Policy decisions regarding adaptation are also made more complex by the uncertainties in the METSSI projections with different models^43^. We consider adaptation in the perfect sense, and therefore our two loss estimates provide an upper and lower bound for future windstorm impacts. Despite this, our results provide a clear benefit indicating where impacts are likely to change through the 21st century, with results acting as a baseline for further investigation by loss modellers and future high-resolution studies.

While the serial clustering of extratropical cyclones is an important contribution to the seasonal losses from European windstorms^1,48^, it is not clear whether this will impact the future of these losses. There are large uncertainties in the projections of storm clustering^49^. Despite this and other uncertainties associated with future changes in the storm tracks and storm intensities^10,50,51^, we are able to give robust projections of changes to the windstorm risk. This does, of course, depend strongly on the population changes that we will see in the coming years. Previous studies have shown that individual models can show non-linear future changes in the storm tracks^52^ with different warming scenarios, and projections from different models depend on a lot of different processes. By using a multi-model ensemble, we show a robust increase in losses in the north of Europe in SSP5-8.5. This highlights that, as well as adapting to future wind speeds, efforts must be in place to follow a lower emissions trajectory to reduce future risk. We have shown that to understand the future changing risk associated with European windstorms, there is a need to go beyond physical hazard modelling to consider risk and adaptation from a socio-economic perspective.

## Methods

### Data

We use the latest reanalysis product from the European Centre for Medium-Range Weather Forecasts (ECMWF), ERA5^53^. Six-hourly data from 1980–2010 are used to identify the extratropical cyclones. The fields used are 850-hPa zonal and meridional winds and 10-m winds. Model data come from 8 models taking part in the latest Coupled Model Intercomparison Project (CMIP6; see Table 1 for details). These models were selected as they provided the data necessary to perform the objective feature tracking for historical and future scenarios. The period used for the historical simulations is 1980–2010, and for SSP2-4.5 and SSP5-8.5, the period of 2070–2100 is used. The focus is only on winter cyclones, so data is restricted to the December-January-February (DJF) season. The first ensemble member (r1i1p1f1 or lowest available) was used where there were multiple members available.

**Table 1:** Details of the CMIP6 models used in this study, including the model name, modelling centre, horizontal and vertical resolution

Model	Centre	Horizontal resolution	Vertical resolution
ACCESS-CM2	CSIRO-ARCCSS; Commonwealth Scientific and Industrial Research Organisation, Australian Research Council Centre of Excellence for Climate System Science, Australia	N96; 192 × 144; 250 km	85 levels to 85 km
BCC-CSM2-MR	BCC; Beijing Climate Center, China	T206; 320 × 160; 100 km	46 levels to 1.46 hPa
EC-Earth3	EC-Earth Consortium	TL255; 512 × 256; 100 km	91 levels to 0.01 hPa
KIOST-ESM	Korea Institute of Ocean Science & Technology Earth System Model	C48; 192 × 96; 250 km	32 levels to 2 hPa
MIROC6	MIROC; MIROC Consortium (JAMSTEC, AORI, NIES, R-CCS), Japan	T85; 256 × 128; 250 km	81 levels to 0.004 hPa
MPI-ESM1.2-HR	MPI-M, DWD, DKRZ; Max Planck Institute for Meteorology, Deutscher Wetterdienst, Deutsches Klimarechenzentrum, Germany	T127; 384 × 192; 100 km	95 levels to 0.01 hPa
MPI-ESM1.2-LR	MPI-M, AWI; Max Planck Institute for Meteorology, Alfred Wegener Institute, Germany	T63; 192 × 96; 250 km	47 levels to 0.01 hPa
MRI-ESM2-0	MRI; Meteorological Research Institute, Japan	TL159; 320 × 160; 100 km	80 levels to 0.01 hPa

All analyses were conducted on a core rectilinear grid defined by longitudinal and latitudinal domains of 23.5°W–31°E and 35°N–71°N respectively, and repeated for three regional sub-domains. The sub-domains are defined as NW Europe (45°N–71°N, 23.5°W–13°E), NE Europe (45°N–71°N, 13°E–31°E), and S Europe (35°N–45°N, 23.5°W–31°E). Prior to any analysis taking place, all data were first re-gridded to a 1.875° × 1.875° grid, which is equivalent to the model with the coarsest resolution. To maintain an exclusive focus on statistics over inhabited land, grid points are masked prior to analysis if their re-gridded human population value is less than one person per square km.

### Cyclone tracking

Storm tracks are identified using a Lagrangian objective cyclone tracking algorithm based on the method of Hodges^54,55^ applied to 850-hPa vorticity. The specific values of all parameters employed within the algorithm, and the methodological details, are identical to those outlined in refs. ^1,38^. The 850-hPa relative vorticity is smoothed to T42 resolution to focus on the synoptic scales before the identification and tracking are applied. From the resulting track lists, only those of a sufficient lifetime (>48 h) and displacement from the point of cyclogenesis (>1000 km), and with at least one track point within the ‘All Europe’ rectilinear domain are retained for analysis.

### Storm footprints

All near-surface wind speed values that occur within 5° of track positions up to 12 h ahead of and behind the passing track are assumed to be attributable to the cyclone in question following the method of ref. ^4^. The maximum of these values within the 24-h period at each location is then used. This area around the cyclone is chosen as it is the area within which the strongest windspeeds tend to occur^27,56^, while the temporal range maximises the proportion of cyclone-attributable wind speeds that are accounted for. This step is used to produce a ‘footprint’ map of maximum cyclonic wind speed values recorded across the domain (Fig. 3).

### Population data

Population density data were derived from population distribution data obtained via the NASA Socio-economic Data and Applications Center (SEDAC)^44,57^. The future projections of population distribution are based on the same Shared Socio-economic Pathways (SSPs) as those used for the storm data. The population base year (2000; Fig. 6a shows the scaled value of the population density) and projection year (2090) applied occur at roughly the mid-points of each respective period covered in the study and were thus assumed to represent good proxies for real insured property values in each respective period. The differences between 2090 and 2000 for the scaled density are shown for SSP2-4.5 and SSP5-8.5 in Fig. 6b and c, respectively.

### Storm severity indices

The SSI is applied as in ref. ^1^ and uses the grid point 98th percentile of the 6-hourly wind speeds as the threshold above which damage occurs. Prior to calculating storm severity indices, we adjusted all local 98th percentile wind speed values below a threshold of 9 m/s up to the value of this threshold, consistent with the methodology used in ref. ^58^. This step ensures that cyclonic wind speeds below the selected threshold do not contribute to the loss metrics. For the SSI in future climatic scenarios, the case of adaptation is considered when the scenario 98th percentile (2070–2099) is greater than the historical value. In this case, the 98th percentile is adjusted upwards to the future scenario value. In cases when the 98^th^ percentile is lower in the future scenario, the historical value is retained as it is assumed that buildings/exposure do not de-adapt. The METSSI is calculated at each grid box as follows:

1 $${{{{{{{\mathrm{METSSI}}}}}}}}_{i,j}={\left(\frac{{v}_{i,j}^{\max }}{{v}_{i,j}^{98}}-1\right)}^{3}$$

2 $${{{{{{\mathrm{MET}}}}}}}{{{{{{{\mathrm{SSI}}}}}}}}_{{{{{{{\mathrm{total}}}}}}}}=\mathop{\sum }\limits_{i=1}^{{N}_{i}}\mathop{\sum }\limits_{j=1}^{{N}_{j}}\,{{{{{{\mathrm{MET}}}}}}}{{{{{{{\mathrm{SSI}}}}}}}}_{i,j}$$

where METSSI is the meteorological storm severity index, *i* and *j* are the spatial co-ordinates of the grid point of interest, *v*^max^ is the maximum wind speed value associated with the cyclone, *v*^98^ is the adjusted 98th percentile value of the winter wind speed distribution, and *N* is the number of grid points within the domain of interest^19,21,30^.

Since the losses associated with windstorms will be strongly related to the exposure of people and buildings, the METSSI can be scaled by the population estimate at each grid box^1,59^.

3 $${{{{{{\mathrm{SOC}}}}}}}{{{{{{{\mathrm{SSI}}}}}}}}_{i,j}={{{{{{{\mathrm{METSSI}}}}}}}}_{i,j}\times {{{{{{{\mathrm{pop}}}}}}}}_{i,j}$$

4 $${{{{{{\mathrm{SOC}}}}}}}{{{{{{{\mathrm{SSI}}}}}}}}_{{{{{{{\mathrm{total}}}}}}}}=\mathop{\sum }\limits_{i=1}^{{N}_{i}}\mathop{\sum }\limits_{j=1}^{{N}_{j}}{{{{{{\mathrm{SOC}}}}}}}{{{{{{{\mathrm{SSI}}}}}}}}_{i,j}$$

Where SOCSSI is the sociological storm severity index, and pop is the human population. For plotting, these values are scaled by 10^−2^ and 10^2^, respectively, to ensure both metrics are of the same order of magnitude.

### Supplementary information

Supplementary Information

## Data Availability

The ERA5 data were available from the Copernicus data store, https://cds.climate.copernicus.eu/cdsapp#!/dataset/reanalysis-era5-pressure-levels?tab=overview, and the CMIP6 model data were available from the Earth System Grid Federation. The generated storm footprints from the current study are available in the GitHub repository, https://github.com/alexslittle/cyclonic-wind-impacts, along with instructions to generate these from the cyclone tracks and the wind speeds.

## Code availability

The objective feature tracking code belongs to Kevin Hodges and is available from the GitLab repository, https://gitlab.act.reading.ac.uk/track/track. The code to calculate the storm footprints is available from the GitHub repository, https://github.com/alexslittle/cyclonic-wind-impacts.
