# Supplementary material for: Future increased risk from extratropical windstorms in northern Europe
Source: Nat Commun. 2023 Jul 22;14:4434. doi: 10.1038/s41467-023-40102-6 (PMC10363171; doi:10.1038/s41467-023-40102-6)
Supplement: Supplementary file 1 — Supplementary Information [file 41467_2023_40102_MOESM1_ESM.pdf]

# Supplementary Material for Future increased risk from extratropical windstorms in northern Europe

Alexander S Little, Matthew D K Priestley, Jennifer L Catto*

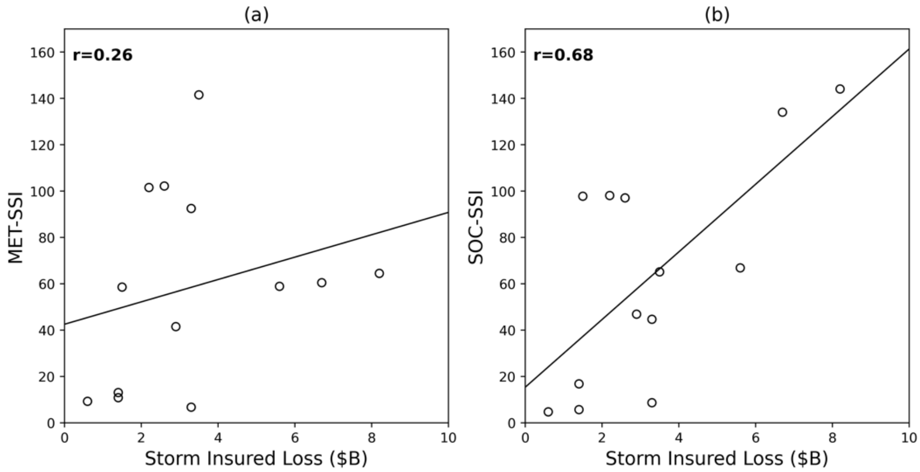


**Fig. S1: Validation of the METSSI (a) and SOCSSI (b) against reported storm losses.** Shown are the normalised METSSI and SOCSSI for the top 13 storms from the XWS dataset calculated with the method described here from ERA5 data against the reported insured losses.

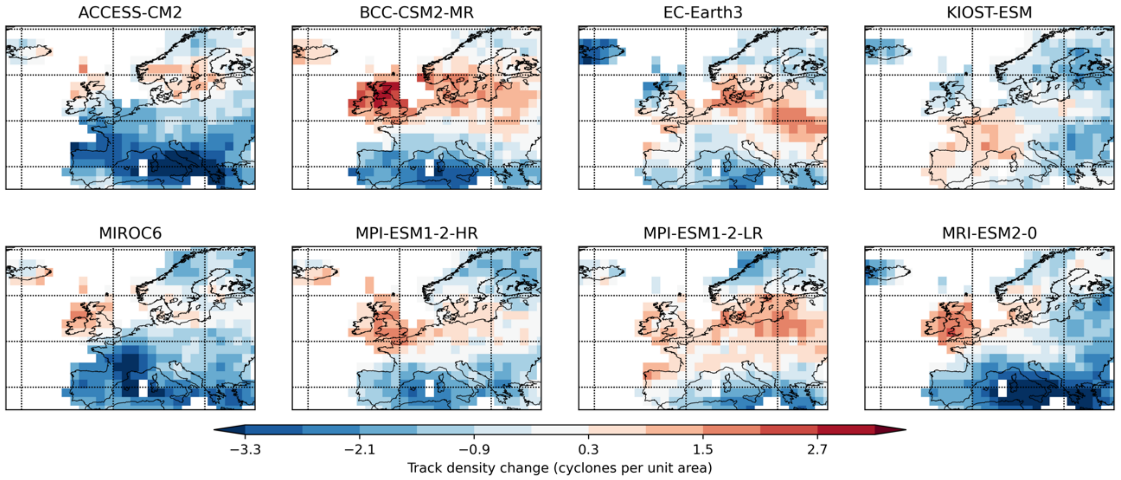


**Fig. S2:** Changes in track density in the SSP5-8.5 scenario (2070-2100) relative to historical (1980-2010) for the 8 CMIP6 models analysed. Units are cyclones per month per unit area.

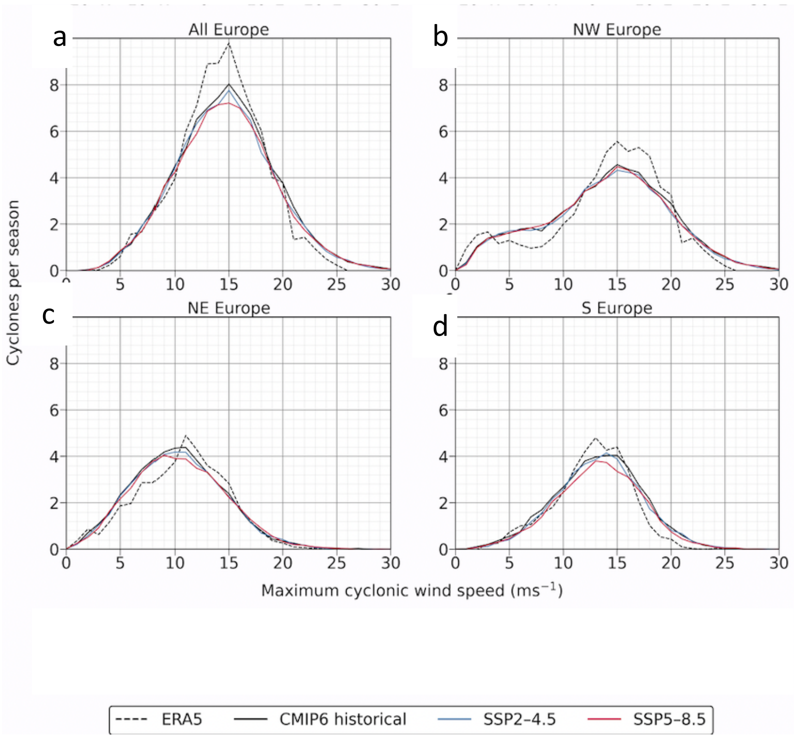


**Fig. S3: Maximum cyclonic windspeeds.** The frequency distributions of maximum cyclonic wind speeds within each cyclone in the four regions (a) All of Europe, (b) NW Europe, (c) NE Europe, and (d) S Europe. Distributions are shown for ERA5, the CMIP6 historical simulations, and the two future scenarios, SSP2-4.5 and SSP5-8.5.

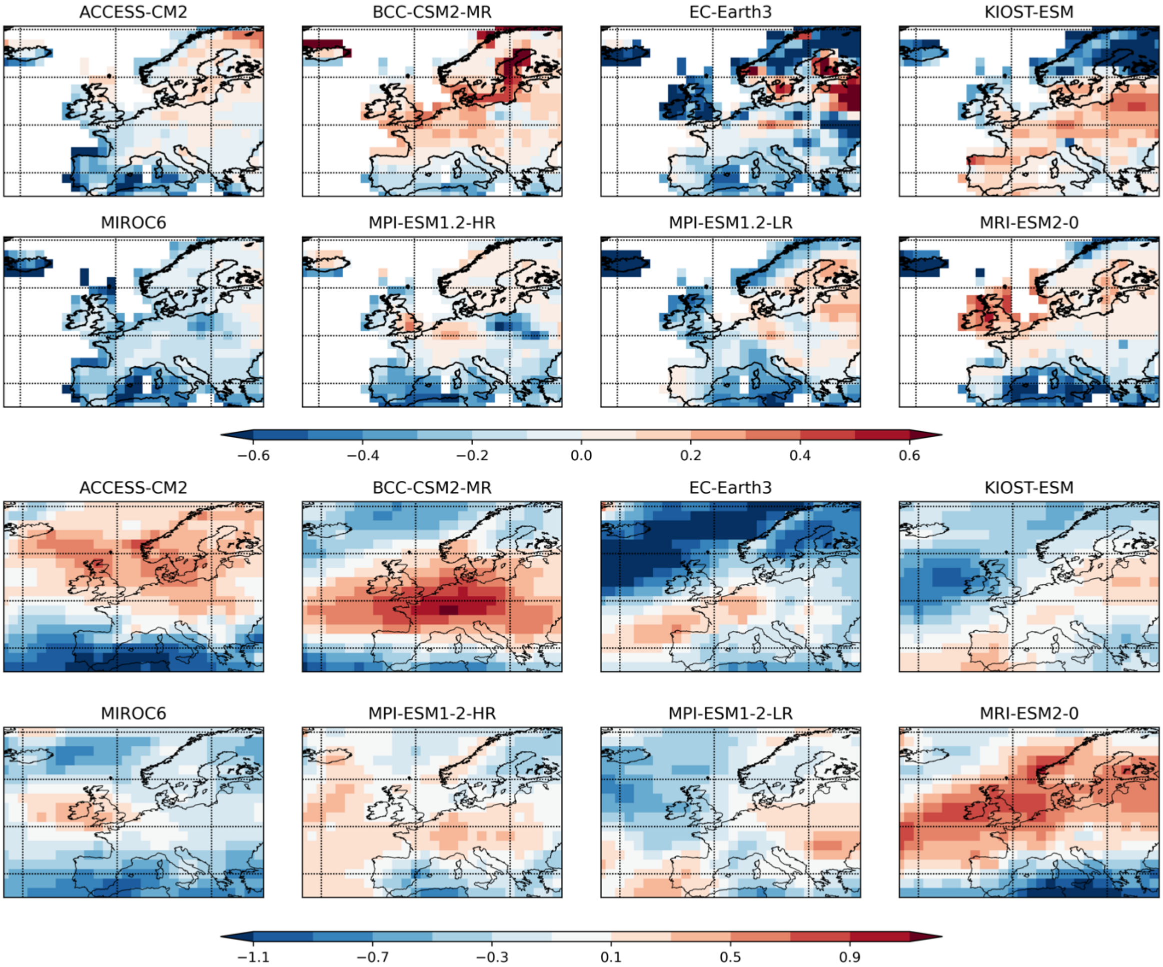


**Fig. S4: Evidence for non-effect of surface processes.** Top set of panels show the future changes of 10-m wind speed in the SSP5-8.5 scenario (2070-2100) for each of the models relative to historical (1980-2010). Wind speed has been calculated using 6-hourly u and v components. Bottom set of panels show the same but for the 850-hPa wind speed. Units are m/s. Similarity of the changes in 850-hPa winds and near-surface winds indicate there are no spurious surface processes affecting the wind speeds.

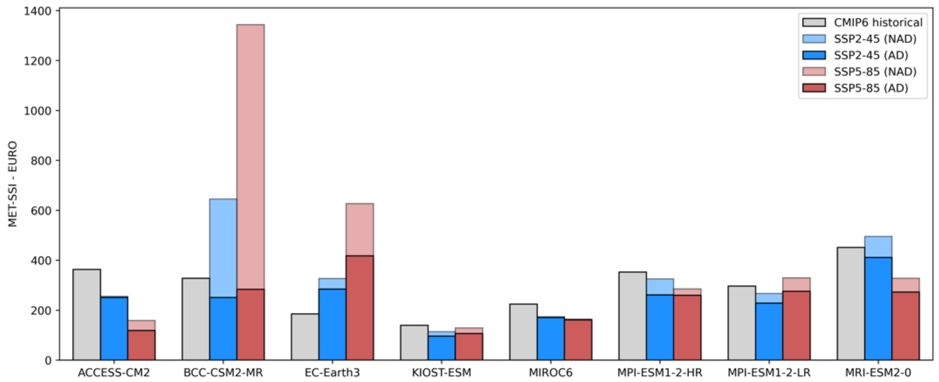


**Fig S5:** Bar chart showing the METSSI for the CMIP6 historical, SSP2-4.5, and SSP5-8.5 simulations for Europe as a whole. The full bars for the future simulations indicate the METSSI when the historical 98th percentile of wind speed is used as the threshold (no adaptation case). The darker bars show the case when the 98th percentile threshold is taken from the corresponding scenario (adaptation case).

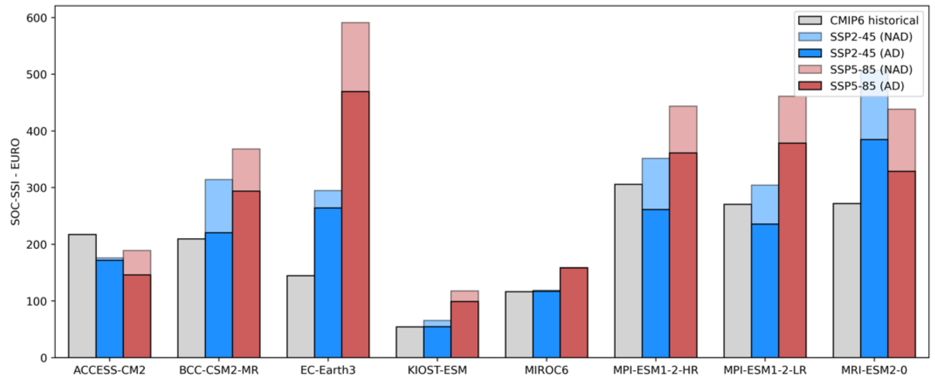


**Fig S6:** Bar chart showing the SOCSSI for the CMIP6 historical, SSP2-4.5, and SSP5-8.5 simulations for Europe as a whole. The full bars for the future simulations indicate the METSSI when the historical 98th percentile of wind speed is used as the threshold (no adaptation case). The darker bars show the case when the 98th percentile threshold is taken from the corresponding scenario (adaptation case).

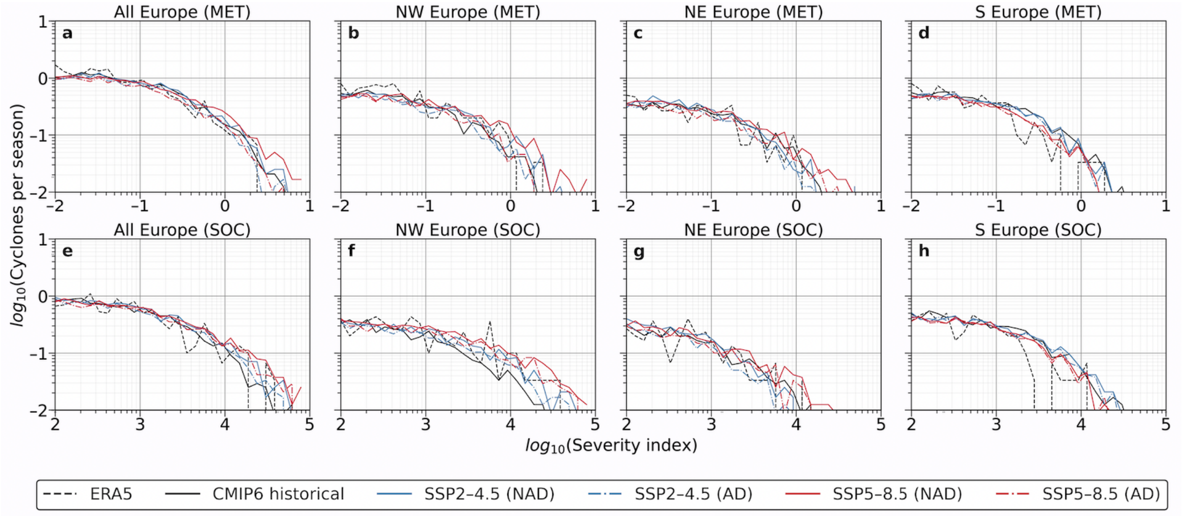


**Fig. S7: Distributions of METSSI and SOCSSI values.** The log-scaled frequency distributions of METSSI (top row), and SOCSSI (bottom row) for all the scenarios.

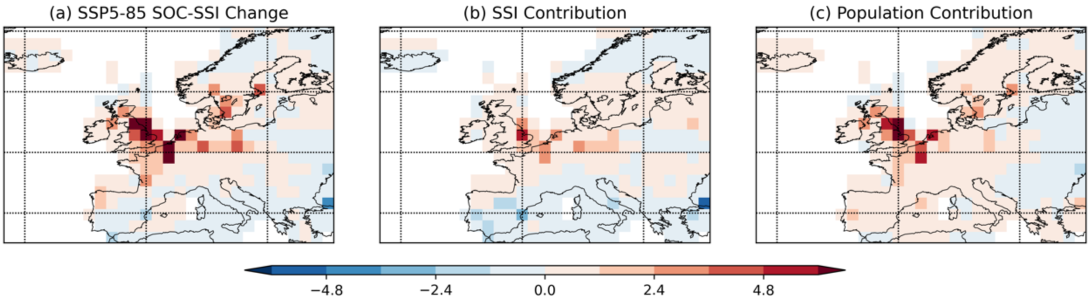


**Fig. S8: Contributions to SOCSSI from storm severity and population.** (a) The map of SOCSSI change without adaptation. (b) The contribution to the SOCSSI from the METSSI component (METSSI multiplied by present day population). (c) The contribution to the SOCSSI from population change at the end of the century for the SSP5-8.5 scenario.
